# Effect of Smoking on Circulating Angiogenic Factors in High Risk Pregnancies

**DOI:** 10.1371/journal.pone.0013270

**Published:** 2010-10-12

**Authors:** Arun Jeyabalan, Robert W. Powers, Rebecca G. Clifton, Peter Van Dorsten, John C. Hauth, Mark A. Klebanoff, Marshall D. Lindheimer, Baha Sibai, Mark Landon, Menachem Miodovnik, for the Eunice Kennedy Shriver National Institute of Child Health and Human Development Maternal-Fetal Medicine Units Network

**Affiliations:** 1 Department of Obstetrics, Gynecology and Women's Health, University of Pittsburgh School of Medicine, Pittsburgh, Pennsylvania, United States of America; 2 The George Washington University Biostatistics Center, Washington, D.C., United States of America; 3 Department of Obstetrics and Gynecology, Medical University of South Carolina, Charleston, South Carolina, United States of America; 4 Department of Obstetrics and Gynecology, University of Alabama at Birmingham School of Medicine, Birmingham, Alabama, United States of America; 5 Eunice Kennedy Shriver National Institute of Child Health and Human Development, Bethesda, Maryland, United States of America; 6 Department of Obstetrics and Gynecology, University of Chicago School of Medicine, Chicago, Illinois, United States of America; 7 Department of Obstetrics and Gynecology, University of Tennessee School of Medicine, Memphis, Tennessee, United States of America; 8 Department of Obstetrics and Gynecology, The Ohio State University School of Medicine, Columbus, Ohio, United States of America; 9 Department of Obstetrics and Gynecology, University of Cincinnati School of Medicine, Cincinnati, Ohio, United States of America; Institute of Zoology, Chinese Academy of Sciences, China

## Abstract

**Objective:**

Changes in maternal concentrations of the anti-angiogenic factors, soluble fms-like tyrosine kinase 1 (sFlt1) and soluble endoglin (sEng), and the pro-angiogenic placental growth factor (PlGF) precede the development of preeclampsia in healthy women. The risk of preeclampsia is reduced in women who smoke during pregnancy. The objective of this study was to investigate whether smoking affects concentrations of angiogenic factors (sFlt1, PlGF, and sEng) in women at high risk for developing preeclampsia.

**Study Design:**

We performed a secondary analysis of serum samples from 993 high-risk women (chronic hypertension, diabetes, multifetal gestation, and previous preeclampsia) in a preeclampsia prevention trial. sFlt1, sEng and PlGF were measured in serum samples obtained at study entry, which was prior to initiation of aspirin (median 19.0 weeks' [interquartile range of 16.0–22.6 weeks']). Smoking status was determined by self-report.

**Results:**

sFlt1 was not significantly different in smokers from any high-risk groups compared to their nonsmoking counterparts. PlGF was higher among smokers compared to nonsmokers among diabetic women (142.7 [77.4–337.3] vs 95.9 [48.5–180.7] pg/ml, p = 0.005) and women with a history of preeclampsia (252.2 [137.1–486.0] vs 152.2 [73.6–253.7] pg/ml, p = 0.001). sEng was lower in smokers with multifetal gestations (5.8 [4.6–6.5] vs 6.8 [5.5–8.7] ng/ml, p = 0.002) and trended lower among smokers with diabetes (4.9 [3.8–5.6] vs 5.3 [4.3–6.3] ng/ml, p = 0.05). Smoking was not associated with a lower incidence of preeclampsia in any of these groups.

**Conclusions:**

In certain high-risk groups, smoking is associated with changes in the concentrations of these factors towards a pro-angiogenic direction during early pregnancy; however, there was no apparent association between smoking and the development of preeclampsia in our cohort.

## Introduction

Cigarette smoke exposure is associated with many adverse pregnancy outcomes including preterm labor, preterm premature rupture of membranes, placental abruption and fetal growth restriction/low birth weight [1], [2]. Paradoxically, smoking is associated with a reduced risk of developing preeclampsia [3], [4]. The mechanism(s) underlying this relationship remains unclear, but one mechanism postulated is that smoking reduces circulating levels of the anti-angiogenic protein soluble fms-like tyrosine kinase (sFlt1).

An imbalance of circulating angiogenic factors, specifically an increase in anti-angiogenic proteins, sFlt1 and sEng, and a decrease in the pro-angiogenic protein, placental growth factor (PlGF), has been proposed as causing some of the clinical manifestations of preeclampsia [5], [6]. Levine et al. have reported significantly lower PlGF and significantly higher sFlt1 and sEng with preeclampsia, even predating its clinical manifestations [7], [8].

Cigarette smoking also influences angiogenic factors with lower circulating sFlt1 concentrations observed in nonpregnant and low risk pregnant smokers [8]–[13]. Findings regarding the relationship between smoking and maternal PlGF are inconsistent with some studies demonstrating higher PlGF in pregnant smokers and others showing no difference [8], [11], [13]. Data regarding sEng are scant with one study suggesting that sEng concentrations may be lower among healthy pregnant smokers [8].

The observations described above focus on low risk pregnancies. There are even fewer data relating to women at high risk for developing preeclampsia. We hypothesized that cigarette smoking during pregnancy would be associated with a pro-angiogenic milieu (lower sFlt1 and sEng as well as higher PlGF) in the maternal circulation of women at high risk for preeclampsia (i.e., pregestational diabetes, chronic hypertension, multifetal gestation, or previous preeclampsia). We also sought to determine the relationship of smoking and the development of preeclampsia in each of these groups.

## Methods

### Study population

We performed a secondary analysis of the Maternal-Fetal Medicine Units Network multicenter randomized controlled trial of low-dose aspirin for the prevention of preeclampsia in high-risk women [14]. A total of 2,539 subjects from 13 participating centers were enrolled between 1991 and 1995 and categorized in four groups: those with pregestational diabetes, chronic hypertension, history of preeclampsia in a previous pregnancy, or multifetal gestation. Subjects were included in the current analysis if they had a study entry serum sample available.

High risk groups were defined as in the primary study [14]. Pregestational diabetics were classified based on insulin treatment prior to pregnancy. The diagnosis of chronic hypertension required medical record documentation of antihypertensive drug therapy or a blood pressure measured in the sitting position equal to or greater than 140/90 mmHg taken on two occasions at least four hours apart, either before conception or during pregnancy but prior to study entry and before gestational week 20. Previous preeclampsia was defined as new-onset proteinuric hypertension after midgestation as determined by medical records or, in the absence of a record, a history of preeclampsia that resulted in delivery before the 37^th^ gestational week. Multifetal gestation required documentation by ultrasound examination before enrollment.

At the screening visit, all women underwent urinary protein testing by dipstick. A 24 hour urine collection was obtained if a value of 1+ or greater was detected. Abnormal proteinuria was diagnosed when total protein excretion was ≥300 mg over 24 hours. Women with multifetal gestations were ineligible for the study if they also had diabetes mellitus, chronic hypertension, or proteinuria as defined above, as were women with a history of preeclampsia and current proteinuria. Women with both diabetes and hypertension were included in the diabetes group.

The diagnosis of preeclampsia was made as per the definitions in the original study [14]. To ensure the validity of consistency in the diagnosis of preeclampsia, the records of all women with apparent preeclampsia in the original trial were independently reviewed by three physicians unaware of treatment group assignments (aspirin vs. placebo) and had to agree unanimously on the validity of the designated outcome.

Subjects had provided written informed consent prior to enrolling in the initial study. This investigation is a companion to a study of the relationship between angiogenic factors and the risk of preeclampsia in these groups [15]. Institutional Review Board approval for the use of these stored samples was obtained at both the University of Pittsburgh and George Washington University. Laboratory assessment was completed at the Magee-Womens Research Institute by investigators blinded to gestational age and high risk category. Data was subsequently linked and statistical analysis completed at the George Washington University Biostatistics Center.

### Determination of smoking status

Smoking status was determined through self-reporting at the time of enrollment and/or sample collection. Subjects were asked whether they had smoked cigarettes in the year prior to pregnancy, whether they quit at the start of pregnancy, and if they continued to smoke, whether they smoked more than a pack of cigarettes per day. Serum cotinine concentrations were available on a subset of the women.

### Laboratory methods

Serum samples were assayed concurrently for sFlt1, sEng and PlGF using commercially available immunoassay kits and each sample run in duplicate according to the manufacturer's protocol (R&D Systems,Minneapolis, MN). All kits for each analyte were from the same manufacturing lot in order to minimize variability. Sensitivities for sFlt1, sEng, and PlGF were 0.005 ng/ml, 0.007 ng/ml, and 7 pg/ml, respectively. Of note, the sFlt assay is specific for human sFlt1 and the PlGF assay does not cross react with VEGF. In general, samples were diluted 1 to 5 for sFlt1 and sEng, and 1 to 2 for PlGF in order for the samples to fall within the measurable range of the kit's standard curves. A small number of samples needed to be re-analyzed because calculated values were outside of the standard curve's range. The inter-assay variability was 10% for sFlt1, 11% for sEng and 7% for PlGF.

### Statistical analysis

Data from each group were analyzed separately. Continuous variables were compared using the Wilcoxon rank-sum test and categorical variables using the chi-square test. Bivariate analysis and multivariable logistic regression were used to determine the association of smoking with the development of preeclampsia. All p-values are two-tailed and a value of less than 0.05 was considered statistically significant. No adjustments were made for multiple comparisons. Analyses were performed using SAS software.

## Results

A total of 993 serum samples obtained at study entry (diabetes, n = 194; chronic hypertension, n = 313; multifetal gestation, n = 234; and previous preeclampsia, n = 252) were available for analysis. The original trial included 2,539 women so this analysis represents 39% of the women. Within each of the groups, women in this analysis (i.e. women with a serum sample at study entry) were similar to women not included (i.e. without a sample) for the following characteristics: primigravid status, smoking, maternal age and body mass index. Compared to women without samples, there were slightly more Caucasians in the diabetes and multifetal groups. The gestational age at study entry was also slightly earlier compared to women without samples in the multifetal group.

Baseline demographic and clinical characteristics for each of the four groups (pregestational diabetes, chronic hypertension, prior preeclampsia, and multifetal gestation) are presented in **Table 1**. Median gestational age at baseline sample collection was 19.0 weeks (interquartile range of 16.0–22.6). Overall, 163 women (16.4%) reported that they were smokers at the time of the baseline interview and 2.2% reported smoking one or more packs of cigarettes per day. The percentage of smokers among diabetics, chronic hypertensives, previous preeclamptics, and women with multifetal gestations were 20%, 17%, 16%, and 13%, respectively.

**Table 1:** Baseline characteristics of women at high risk for preeclampsia.*

Characteristics	Pregestational diabetes (n = 194)			Chronic hypertension (n = 313)			Previous preeclampsia (n = 252)			Multifetal gestation (n = 234)		
	Smoker (n = 39)	Nonsmoker (n = 155)	p-value	Smoker (n = 53)	Nonsmoker (n = 260)	p-value	Smoker (n = 40)	Nonsmoker (n = 212)	p-value	Smoker (n = 31)	Nonsmoker (n = 203)	p-value
**Maternal age (years)**	26±6	26±6	0.64	30±7	29±7	0.18	26±5	24±5	0.02	26±5	25±6	0.21
**Gestational age at entry (weeks)**	18±3	17±4	0.16	19±4	19±4	0.73	20±4	20±4	0.41	21±4	21±4	0.44
**Primigravida, n (%)**	6 (15)	60 (39)	<0.01	7 (13)	58 (22)	0.14	0 (0)	0 (0)	n/a	6 (19)	66 (33)	0.14
**Race or ethnic group, n (%)**			0.47			0.07			0.03			<0.01
**Caucasian**	27 (69)	91 (59)		17 (32)	66 (25)		15 (38)	40 (19)		19 (61)	73 (36)	
**African-American**	10 (26)	51 (33)		35 (66)	162 (62)		24 (60)	166 (78)		12 (39)	102 (50)	
**Hispanic/other**	2 (5)	13 (8)		1 (2)	32 (12)		1 (3)	6 (3)		0 (0)	28 (14)	
**Systolic blood pressure at entry (mmHg)**	114±14	114±15	0.98	121±14	129±14	<0.01	113±13	111±11	0.37	110±10	112±11	0.29
**Diastolic blood pressure at entry (mmHg)**	68±10	68±10	0.73	70±13	78±11	<0.01	64±11	66±9	0.50	62±10	64±9	0.23
**Body mass index (kg/m^2^)**	27±7	28±8	0.54	31±7	34±9	0.03	28±8	28±8	0.86	26±7	27±7	0.51

*Data are presented as mean ± standard deviation or n (%).

We compared sFlt1, sEng, and PlGF concentrations between pregnant smokers and nonsmokers in each of the four groups separately (**Tables 2** **–** [Table pone-0013270-t003] [Table pone-0013270-t004] **5**). Women with diabetes who smoked had higher circulating PlGF (142.7 [77.4–337.3] vs 95.9 [48.5–180.7], p = 0.005), slightly lower soluble endoglin (4.9 [3.8–5.6] vs 5.3 [4.3–6.3], p = 0.05), and no difference in sFlt1 than those who did not smoke. Among women with chronic hypertension, there were no differences between smokers and nonsmokers in the three angiogenic factors measured. Among women with preeclampsia in a prior pregnancy, only PlGF was significantly higher in smokers than in nonsmokers (252.2 [137.1–486.0] vs 152.2 [73.6–253.7], p = 0.001). Among women with multifetal gestations, only sEng was different (i.e., lower) in smokers compared to nonsmokers.

**Table 2:** Comparison of angiogenic factors in pregnant women with pregestational diabetes: smokers versus nonsmokers.*

Pregestational diabetes			
	Smoker (n = 39)	Nonsmoker (n = 155)	p-value
**sFlt1 (ng/ml)**	3.0 (2.5–4.2)	3.3 (2.2–4.4)	0.99
**sEng (ng/ml)**	4.9 (3.8–5.6)	5.3 (4.3–6.3)	0.05
**PlGF (pg/ml)**	142.7 (77.4–337.3)	95.9 (48.5–180.7)	0.005

*Data are presented as median (inter-quartile range).

**Table 3 pone-0013270-t003:** Comparison of angiogenic factors in pregnant women with chronic hypertension: smokers versus nonsmokers.*

Chronic hypertension			
	Smoker (n = 53)	Nonsmoker (n = 260)	p-value
**sFlt1 (ng/ml)**	3.1 (2.4–4.6)	3.0 (2.1–4.3)	0.52
**sEng (ng/ml)**	4.9 (4.3–5.4)	4.8 (4.2–5.9)	0.71
**PlGF (pg/ml)**	157.8 (111.3–260.7)	137.8 (70.9–282.0)	0.22

*Data are presented as median (inter-quartile range).

**Table 4 pone-0013270-t004:** Comparison of angiogenic factors in pregnant women with preeclampsia in a previous pregnancy: smokers versus nonsmokers.*

Previous preeclampsia			
	Smoker (n = 40)	Nonsmoker (n = 212)	p-value
**sFlt1 (ng/ml)**	2.6 (1.9–3.6)	3.1 (2.2–4.0)	0.10
**sEng (ng/ml)**	4.6 (4.0–5.4)	5.0 (4.0–5.9)	0.44
**PlGF (pg/ml)**	252.2 (137.1–486.0)	152.2 (73.6–253.7)	0.001

*Data are presented as median (inter-quartile range).

**Table 5:** Comparison of angiogenic factors in women with multifetal gestations: smokers versus nonsmokers.*

Multifetal gestation			
	Smoker (n = 31)	Nonsmoker (n = 203)	p-value
**sFlt1 (ng/ml)**	5.2 (4.2–6.6)	6.0 (4.2–7.7)	0.11
**sEng (ng/ml)**	5.8 (4.6–6.5)	6.8 (5.5–8.7)	0.002
**PlGF (pg/ml)**	491.2 (211.0–728.5)	403.5 (229.2–756.8)	0.40

*Data are presented as median (inter-quartile range).

Given the higher sFlt1 and sEng, as well as lower PlGF concentrations prior to and after recognition of preeclampsia in low risk women, we also analyzed our data by removing women who eventually developed preeclampsia. With the exclusion of 46 diabetic women, 78 chronic hypertensive women, and 39 women with multifetal gestations who developed preeclampsia, there were no changes in the relationship of sFlt1, sEng, or PlGF between smokers and nonsmokers. When the 50 women who developed preeclampsia were excluded from the group with prior preeclampsia, sFlt1 was significantly lower in smokers compared to nonsmokers (2.4 vs 3.1 ng/ml, p = 0.02), but the relationship of PlGF and sEng between smokers and nonsmokers remained the same.

Smoking in early pregnancy was not associated with a reduction in the incidence of preeclampsia in any of the four groups using bivariate analysis and multivariable logistic regression analysis controlling for maternal age, race, BMI and treatment group (aspirin or placebo) (**Table 6**).

**Table 6:** Association of smoking and development of preeclampsia in high risk groups.

	Rate of preeclampsia among smokers, n (%)	Rate of preeclampsia among nonsmokers, n (%)	Odds ratio (95% CI)	Adjusted odds ratio* (95% CI)
**Pregestational diabetes (n = 194)**	5 (12.8)	41 (26.5)	0.41 (0.15–1.12)	0.40 (0.15–1.12)
**Chronic hypertension (n = 313)**	13 (24.5)	65 (25.0)	0.98 (0.49–1.94)	0.97 (0.48–1.95)
**Multifetal gestation (n = 234)**	3 (9.7)	36 (17.7)	0.50 (0.14–1.72)	0.53 (0.15–1.87)
**Previous preeclampsia (n = 252)**	9 (22.5)	41 (19.3)	1.21 (0.54–2.74)	1.17 (0.51–2.71)

*adjusted for maternal age, race, BMI, treatment group (aspirin or placebo).

Although we assessed maternal cigarette smoke exposure by self-report, serum cotinine concentrations from a previous analysis were available for 673 (68%) of the 993 serum samples analyzed. In this cohort, the degree of agreement between self-report of smoking status and a cotinine concentration of ≥10 ng/ml, a cut-off representing active smoking[16], was high (kappa coefficient = 0.81); 95.5% of women who self-reported smoking had cotinine concentrations of ≥10 ng/ml and 94.1% of self-reported nonsmokers had concentrations <10 ng/ml.

## Discussion

We sought to describe the relationship between smoking, angiogenic factors and preeclampsia in four well-defined high risk pregnancy groups. The major findings of our study were (1) maternal sFlt1 concentrations *are not* reduced with smoking, (2) smoking *is* associated with changes in circulating PlGF and sEng concentrations toward a pro-angiogenic direction but these differences were not consistent across all high risk groups, and (3) smoking in early pregnancy *is not* associated with a reduced incidence of preeclampsia.

We measured sFlt1, sEng and PlGF because of their proposed pathologic role in preeclampsia *and* the influence of smoking on their circulating levels during pregnancy. Based on consistent reports of low sFlt1 in nonpregnant and healthy pregnant smokers, we hypothesized and fully expected sFlt1 to be lower among smokers in the high risk groups studied. However, this was not the case in any of the four groups. On the other hand, PlGF was higher in smokers with pregestational diabetes and prior preeclampsia compared to their nonsmoking counterparts and sEng was lower in smokers with multifetal gestations and diabetes. Excluding women who developed preeclampsia did not substantially change our results, except that sFlt1 was lower in smokers with prior preeclampsia compared with nonsmokers. We did not analyze the data based on quantity of cigarette smoking because only 2.2% of women in our cohort smoked greater than one pack per day limiting any meaningful analyses based on dose response of the number of cigarettes smoked and angiogenic factor concentrations. All the significant differences were consistently in a pro-angiogenic direction, i.e., higher PlGF and lower sEng among smokers. While existing data are limited, higher PlGF and lower sEng were also observed among smokers in a low risk population [8], [11]. PlGF concentrations also trended higher in placental villous explants exposed to cigarette smoke extract, lending biologic support for the effect of smoking on PlGF in certain subgroups of our study [17]. Further evidence for the potential pro-angiogenic effects of smoking is that smoking is associated with acceleration of diabetic retinopathy [18].

The lack of consistent findings across the four groups is not surprising as these are pathologically different disorders and their contribution to pregnancy complications such as preeclampsia is likely to be heterogeneous. For example, chronic hypertension is primarily characterized by endothelial dysfunction and microvascular disease whereas with diabetes the pathology may be due to direct effects of hyperglycemia in addition to vascular disease. Furthermore, the maternal versus fetal/placental contribution to circulating angiogenic factors may be quite different, further confounding any superimposed effect of smoking.

In low risk pregnancies, the association between cigarette smoking and a reduced risk of preeclampsia has been a consistent and reproducible finding across epidemiologic studies [3], [4]. Therefore, we expected to find a similar relationship in the high risk groups that we studied. We did not observe a significant association between smoking in early pregnancy and reduced risk of developing preeclampsia in any group. Our negative findings may be attributed to the relatively small numbers in each group; for example, among diabetic women and women with multifetal pregnancies the point estimates are in the direction of a reduced risk of preeclampsia but the confidence intervals are wide (0.40 [95% confidence interval, 0.15–1.12] in diabetics and 0.53 [0.15–1.87] in the multifetal gestation group). A larger sample size may lend more precision to the odds ratios for the relationship between smoking and preeclampsia within each group. However, others have also reported negative findings among various high risk cohorts. Chappell and colleagues failed to find an association between smoking and a lower rate of preeclampsia among chronic hypertensive women and suggested that smoking may even be an independent risk factor for superimposed preeclampsia [19]. The lack of a significant association was also demonstrated among twin pregnancies [20], [21], overweight/obese women [22], African American women [23], and older women [24]. Data regarding smoking and preeclampsia in diabetic pregnancies are variable with no mitigating effect in a large cohort of gestational diabetic women [25], but a risk reduction in two smaller case series of women with pregestational diabetes [26], [27]. An alternative explanation may be that smoking in early pregnancy may not be associated with a lower incidence of preeclampsia. A recent study by Wilkstrom and colleagues demonstrated that there was no significant association of smoking and preeclampsia in women who quit smoking prior to late pregnancy in a Swedish cohort of low risk women [28], [29]. The precise reason as to why smoking may be associated with a lower rate of preeclampsia in low risk but not high risk women is not known, but we speculate that the underlying pathology increases the risk of preeclampsia to such a degree that any measurable effect of smoking is superceded.

As previously mentioned, sample size within each group and the retrospective approach are limitations of our study. Importantly, the inability to assess smoking status across pregnancy and account for changes in smoking patterns may affect circulating angiogenic factors and subsequent risk of preeclampsia. Despite these limitations, our study is novel in addressing the relationship between smoking and angiogenic factors as well as smoking and preeclampsia among groups of women at high risk for developing preeclampsia. A particular strength of this study is that the groups and the diagnostic criteria for preeclampsia are well defined as described in the original Maternal-Fetal Medicine Units Network trial [14]. All samples in this study were analyzed in a blinded fashion. Furthermore, we confirm self-report of smoking with cotinine concentrations in a subpopulation, thereby reducing misclassification bias [30]. Our results do not address mechanistic pathways by which smoking may affect certain angiogenic factors in these groups of women at high risk for preeclampsia. Further study is warranted to better define the biological mechanisms and complex interactions within specific groups and potential reasons for the differences we observed.
